# Immortalized human myotonic dystrophy muscle cell lines to assess therapeutic compounds

**DOI:** 10.1242/dmm.027367

**Published:** 2017-04-01

**Authors:** Ludovic Arandel, Micaela Polay Espinoza, Magdalena Matloka, Audrey Bazinet, Damily De Dea Diniz, Naïra Naouar, Frédérique Rau, Arnaud Jollet, Frédérique Edom-Vovard, Kamel Mamchaoui, Mark Tarnopolsky, Jack Puymirat, Christophe Battail, Anne Boland, Jean-Francois Deleuze, Vincent Mouly, Arnaud F. Klein, Denis Furling

**Affiliations:** 1 Sorbonne Universités UPMC Univ Paris 06, INSERM, CNRS, Centre de Recherche en Myologie, Institut de Myologie, GH Pitié-Salpêtrière, Paris 75013, France; 2 McMaster University Medical Center, Departments of Pediatrics and Medicine, 1200 Main St W., Hamilton, Ontario, Canada, L8N 3Z5; 3 CHU de Quebec, site Enfant-Jésus, Université Laval, Québec, CanadaG1J 1Z4; 4 Centre National de Génotypage, Institut de Génomique, CEA, 91000 Evry, France

**Keywords:** Myotonic dystrophy, Expanded repeats, Muscle cell line, Nuclear aggregates, Alternative splicing, Therapeutic compounds

## Abstract

Myotonic dystrophy type 1 (DM1) and type 2 (DM2) are autosomal dominant neuromuscular diseases caused by microsatellite expansions and belong to the family of RNA-dominant disorders. Availability of cellular models in which the DM mutation is expressed within its natural context is essential to facilitate efforts to identify new therapeutic compounds. Here, we generated immortalized DM1 and DM2 human muscle cell lines that display nuclear RNA aggregates of expanded repeats, a hallmark of myotonic dystrophy. Selected clones of DM1 and DM2 immortalized myoblasts behave as parental primary myoblasts with a reduced fusion capacity of immortalized DM1 myoblasts when compared with control and DM2 cells. Alternative splicing defects were observed in differentiated DM1 muscle cell lines, but not in DM2 lines. Splicing alterations did not result from differentiation delay because similar changes were found in immortalized DM1 transdifferentiated fibroblasts in which myogenic differentiation has been forced by overexpression of MYOD1. As a proof-of-concept, we show that antisense approaches alleviate disease-associated defects, and an RNA-seq analysis confirmed that the vast majority of mis-spliced events in immortalized DM1 muscle cells were affected by antisense treatment, with half of them significantly rescued in treated DM1 cells. Immortalized DM1 muscle cell lines displaying characteristic disease-associated molecular features such as nuclear RNA aggregates and splicing defects can be used as robust readouts for the screening of therapeutic compounds. Therefore, immortalized DM1 and DM2 muscle cell lines represent new models and tools to investigate molecular pathophysiological mechanisms and evaluate the *in vitro* effects of compounds on RNA toxicity associated with myotonic dystrophy mutations.

## Abstract (precis)

**Summary:** Myotonic dystrophy muscle cell models displaying characteristic disease-associated molecular features can be used to investigate molecular pathophysiological mechanisms and evaluate therapeutic approaches.

## INTRODUCTION

Myotonic dystrophy (DM) including both type 1 and type 2 forms is one of the most prevalent muscular dystrophies in adults. Both DM forms are autosomal dominant disorders characterized by myotonia, progressive muscle weakness and wasting, cardiac conduction defects as well as cognitive impairments and endocrine dysfunctions (Harper, 2001). The most common type 1 form (DM1, also called Steinert disease) is caused by the abnormal expansion of a repeated CTG trinucleotide in the 3′UTR of the *DMPK* gene (Brook et al., 1992; Fu et al., 1992; Mahadevan et al., 1992). The size of the expansion can reach more than 4000 CTG repeats in DM1 patients compared with 5-37 CTG repeats in non-affected individuals. The unstable CTG expansion increases over successive generations (Lavedan et al., 1993) and the size of the expanded repeats globally correlates with disease severity (Groh et al., 2011). The type 2 form (DM2) is due to a large CCTG expansion that can reach up to 11,000 repeats in the first intron of the *CNBP* gene (Liquori et al., 2001). Both DM forms share similar clinical features; however, differences exist such as age of onset or pattern of muscle wasting that affects predominantly distal muscles in DM1 and proximal muscles in DM2 (Day and Ranum, 2005). In addition, clinical symptoms are milder in DM2 than in DM1 and, in contrast to DM1, there is no congenital form in DM2.

Myotonic dystrophy is part of a new family of RNA gain-of-function diseases (Klein et al., 2011) and both DM forms share a common pathophysiological feature: expression of mutant RNAs containing expanded C/CUG repeats (C/CUGexp-RNA) that are retained in the nucleus as discrete aggregates (Davis et al., 1997; Liquori et al., 2001; Taneja et al., 1995). In skeletal muscles, nuclear aggregates of C/CUGexp-RNAs sequester the regulatory splicing factor MBNL1 leading to its functional loss and subsequently, to alternative splicing misregulation (Fardaei et al., 2002; Lin et al., 2006; Liquori et al., 2001; Mankodi et al., 2001; Miller et al., 2000). More than 40 mis-splicing events have been confirmed in affected skeletal muscles of DM1 patients (Nakamori et al., 2013); in particular, altered splicing of *CLCN1*, *INSR*, *BIN1* and *DMD* pre-mRNAs (Charlet-B et al., 2002; Savkur et al., 2001; Fugier et al., 2011; Rau et al., 2015) have been associated with myotonia, insulin resistance, muscle weakness and muscle fiber disorganization, respectively, which are all typical symptoms of myotonic dystrophy.

Although animal models including mouse, fly, zebrafish or worm have been developed during the last 15 years to investigate pathophysiologic mechanisms involved in DM1, and despite the fact that several therapeutic strategies are under development (Klein et al., 2015), there is still no cure for DM1 to date. However, it is worth noting that an antisense oligonucleotide (ASO) approach (IONIS-DMPK-2.5Rx) is currently being tested in a Phase 1/2a clinical trial (clinicaltrials.gov, NCT02312011) (Pandey et al., 2015; Wheeler et al., 2012). Mouse models expressing expanded CTG repeats in skeletal muscles have also been used to evaluate the efficacy of therapeutic approaches, including antisense oligonucleotides (ASOs), gene therapies and small molecules, on DM1-associated molecular features such as the presence of nuclear CUGexp-RNA aggregates and alternative splicing misregulation, or muscle dysfunction, such as myotonia (Gomes-Pereira et al., 2011). Nevertheless, there remains a need for cellular models to evaluate compounds *in vitro* to allow middle- or high-throughput screenings, before *in vivo* validation. For this purpose, primary muscle cell cultures derived from muscle biopsies of DM1 patients represent a valuable model since the CTG expansion is expressed within its natural genomic context. Moreover, DM1 muscle cells show assessable DM1-associated molecular features including CUGexp-RNA nuclear aggregates that sequester MBNL1 and subsequent alternative splicing defects (Botta et al., 2013; Dansithong et al., 2005; Francois et al., 2011; Furling et al., 2001; Holt et al., 2007; Loro et al., 2010). However, several difficulties may limit the use of primary muscle cell cultures, especially considering high-throughput screening approaches that require a large number of cells with a reliable and robust phenotype. A major concern is the accessibility and availability of muscle biopsies from DM patients. In addition, the limited proliferative capacity of adult human myoblasts that is inversely correlated to the age of the dystrophic patients constitutes another constraint (Hayflick, 1965; Renault et al., 2000). Furthermore, proliferation of DM primary myoblasts is reduced when compared with age-matched control myoblasts because of the triggering of premature replicative senescence (Bigot et al., 2009; Renna et al., 2014; Thornell et al., 2009). All these limitations support the development of immortalized human DM muscle cell lines in which replicative senescence is bypassed but that display robust and reliable disease-associated features.

Here, we established and characterized novel DM cellular models of human immortalized muscle cell lines. Proof-of-concept using antisense approaches and transcriptomic analyses was performed to validate these immortalized DM muscle cell lines as dependable tools to assess the *in vitro* effects of therapeutic compounds on RNA toxicity associated with DM mutations.

## RESULTS

### Generation of immortalized human DM muscle cell lines

**Fig. 1.**
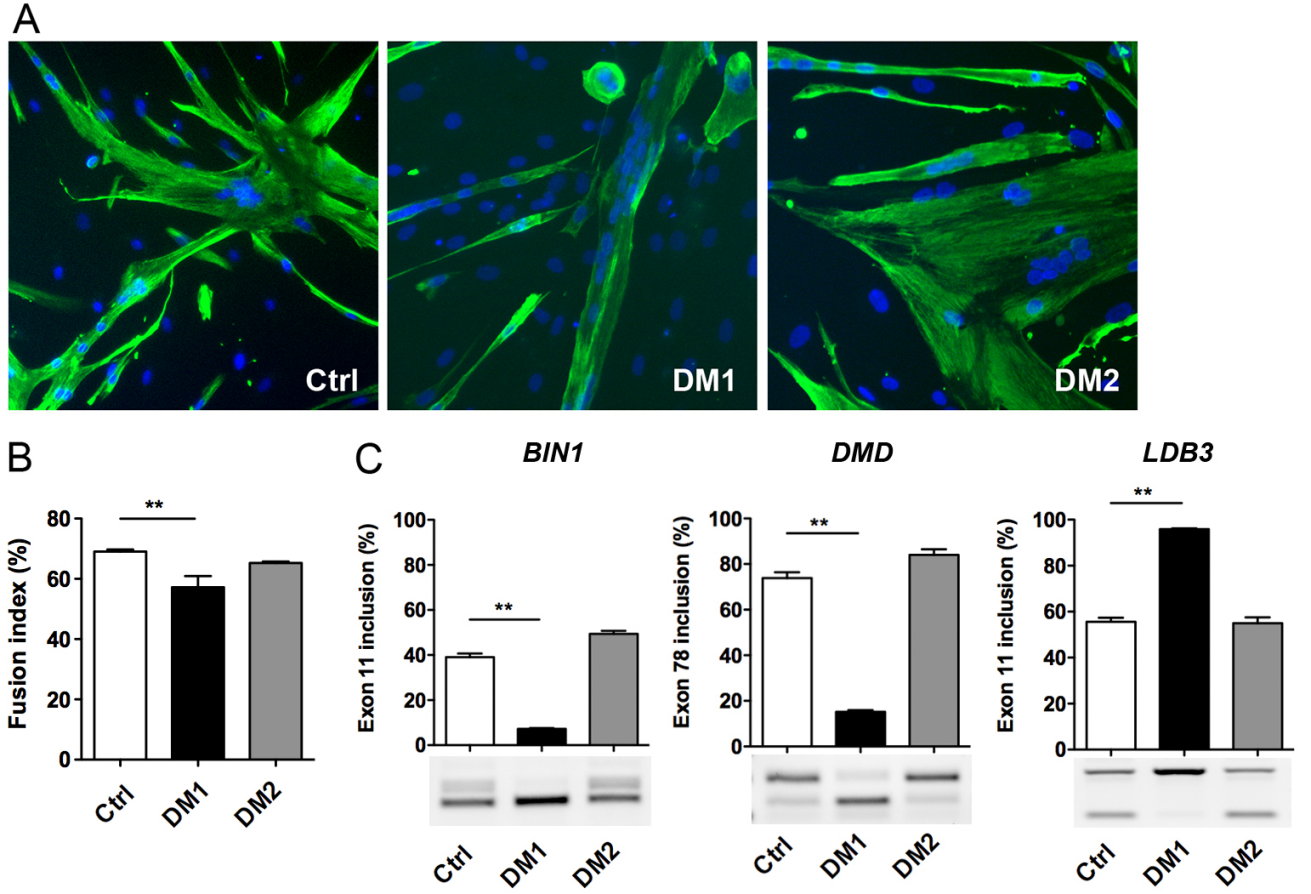
**Features of primary DM muscle cells.** (A) Control (Ctrl), DM1 and DM2 primary myoblasts were differentiated for 4 days and then fixed for immunofluorescence analysis using desmin antibody (green) and nuclei staining (blue) with Hoechst. (B) The fusion index of differentiated primary muscle cell cultures determined by the percentage of the number of nuclei in myotubes (>2 myonuclei) to the total number of nuclei in desmin-positive cells (*n*=4 for Ctrl, *n*=3 for DM1 and DM2, >1000 nuclei counted per experiment). (C) RT-PCR analysis of alternative splicing profile of *BIN1* exon 11, *DMD* exon 78 and *LDB3* exon 11 for differentiated cultures of Ctrl, DM1 and DM2 primary muscle cells (*n*=3 in duplicate for each condition). Data are expressed as mean±s.e.m. Comparison with one-way ANOVA with Newman-Keuls post-test; **P<0.01.

To establish DM myoblast cell lines, primary muscle cell cultures obtained from muscle biopsies of DM patients carrying either a DM1 mutation with 2600 CTG or a DM2 mutation with 4000 CCTG were selected. Characterization of these primary cell cultures showed that DM1 myoblasts form smaller myotubes under differentiation conditions and presented a reduced fusion capacity when compared with non-DM control (Ctrl) myoblasts (Fig. 1A,B). In contrast, the fusion index of differentiated DM2 myoblast cultures was not significantly different from differentiated Ctrl myoblast cultures and the size of the multinucleated DM2 myotubes was comparable to that of Ctrl myotubes, as reported previously for these primary muscle cells (Pelletier et al., 2009). At the molecular level, nuclear RNA aggregates of expanded CUG or CCUG repeats were detected by fluorescent *in situ* hybridization (FISH) in DM1 and DM2 muscle cells, respectively (data not shown), in agreement with previous studies (Furling et al., 2001; Giagnacovo et al., 2012). Alternative splicing changes affecting *BIN1* exon 11, *DMD* exon 78 and *LDB3* exon 11 were also detected in differentiated DM1 muscle cell cultures but not in differentiated DM2 or Ctrl muscle cell cultures (Fig. 1C), in accordance with a previous report in affected muscles of a large cohort of DM1 patients (Nakamori et al., 2013).

**Fig. 2.**
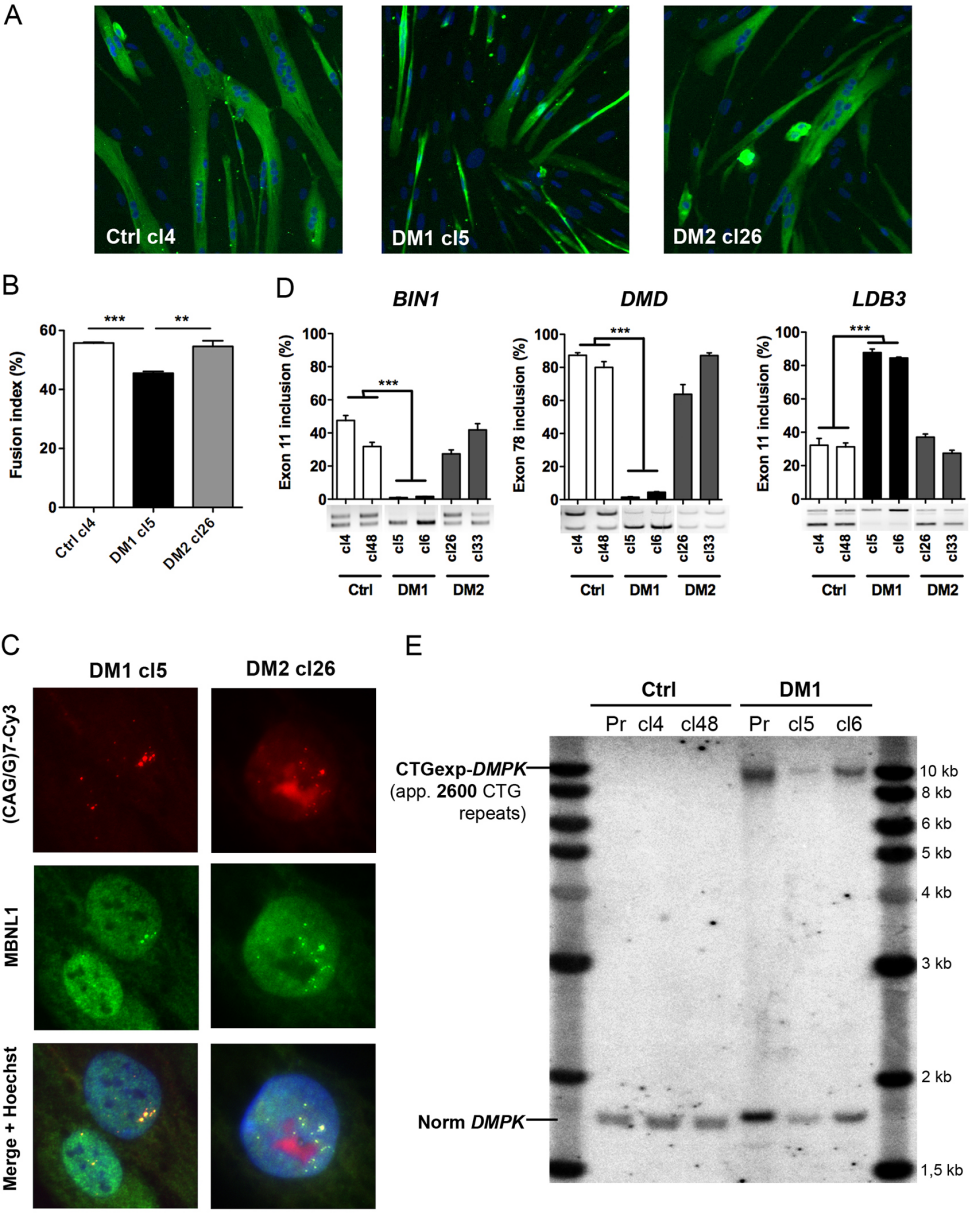
**Characterization of DM myoblast cell lines.** (A) Representative cultures of Ctrl (cl4), DM1 (cl5) and DM2 (cl26) immortalized myoblasts differentiated for 4 days and then fixed for immunofluorescence analysis using desmin antibody (green) and Hoechst nuclear staining (blue). (B) The fusion index of differentiated immortal myoblast cultures was determined by the percentage of the number of nuclei in myotubes (>2 myonuclei) to the total number of nuclei in desmin-positive cells (*n*=3 for each condition, >1000 nuclei counted per experiment). (C) Nuclear RNA aggregates of expanded-CUG or -CCUG repeats in immortalized DM1 and DM2 myoblasts were detected by FISH using a Cy3-(CAG)7 or Cy3-(CCAG)5 probe (red), respectively. (D) Quantification of alternative splicing profile of *BIN1* exon 11, *DMD* exon 78 and *LDB3* exon 11 by RT-PCR analysis in differentiated cultures of Ctrl, DM1 and DM2 immortal myoblast cell lines (*n*=3 for each condition). (E) Southern blot analysis using a *DMPK* probe to determine the size of the CTG expansion in primary DM1 muscle cells (Pr) and two clones of immortalized DM1 myoblasts (Cl5 and Cl6) derived from the same primary muscle cell culture. Data are expressed as mean±s.e.m. Comparison with one-way ANOVA with Newman-Keuls post-test; **P<0.01, ***P<0.001.

For immortalization, primary DM1, DM2 and Ctrl myoblasts were co-transduced with two lentiviral vectors expressing the catalytic subunit of the human telomerase (TERT) and the natural p16 (CDKN2A) ligand, CDK4. Co-transduced cells were then selected as previously described (Zhu et al., 2007; Mamchaoui et al., 2011) and several immortalized myogenic clones were isolated from each population. After testing their ability to differentiate into myotubes, we selected clones showing the highest fusion efficiency, and one clone of each cell line (DM1-cl5, DM2-cl26 and Ctrl-cl4) was further characterized. Under differentiation conditions, immortalized myoblasts behave similarly to the primary myoblast cultures from which they were derived. Thus, DM1-cl5 myoblasts form smaller myotubes than Ctrl-cl4 myoblasts (Fig. 2A) and the fusion index of differentiated DM1-cl5 myoblast cultures was reduced when compared with Ctrl-cl4 myoblast cultures (Fig. 2B). In contrast, DM2-cl26 myotubes were similar to Ctrl-cl4 myotubes and there was no difference between the fusion capacity of differentiated DM2-cl26 and Ctrl-cl4 muscle cell cultures. Then, we investigated whether the molecular hallmarks of DM such as nuclear RNA aggregates and alternative splicing misregulation were found in these muscle cell lines. As expected, FISH and immunofluorescence analyses allowed the detection of nuclear aggregates of CUGexp- and CCUGexp-RNA in immortalized DM1 and DM2 cells, respectively, and the colocalization of MBNL1 with CUGexp- and CCUGexp-RNA foci (Fig. 2C). However the level of CELF1 RNA binding protein that is altered in cells expressing CUGexp-RNA was not significantly changed in either of the immortalized DM1 clones compared with the control (Fig. S1). Furthermore, RT-PCR analysis showed that the splicing pattern of *BIN1* exon 11, *DMD* exon 78 or *LDB3* exon 11 pre-mRNAs was significantly deregulated in differentiated DM1-cl5 or DM1-cl6 muscle cells compared with Ctrl-cl4 or Ctrl-cl48 and DM2-cl26 or DM2-cl33 muscle cells (Fig. 2D). Finally, Southern blot analysis indicated that the size of the expanded CTG repeat (∼2600 CTG), was similar between immortalized DM1 myoblasts (either cl5 or cl6) and the parental primary population of DM1 myoblasts used to establish these cell lines (Fig. 2E).

**Fig. 3.**
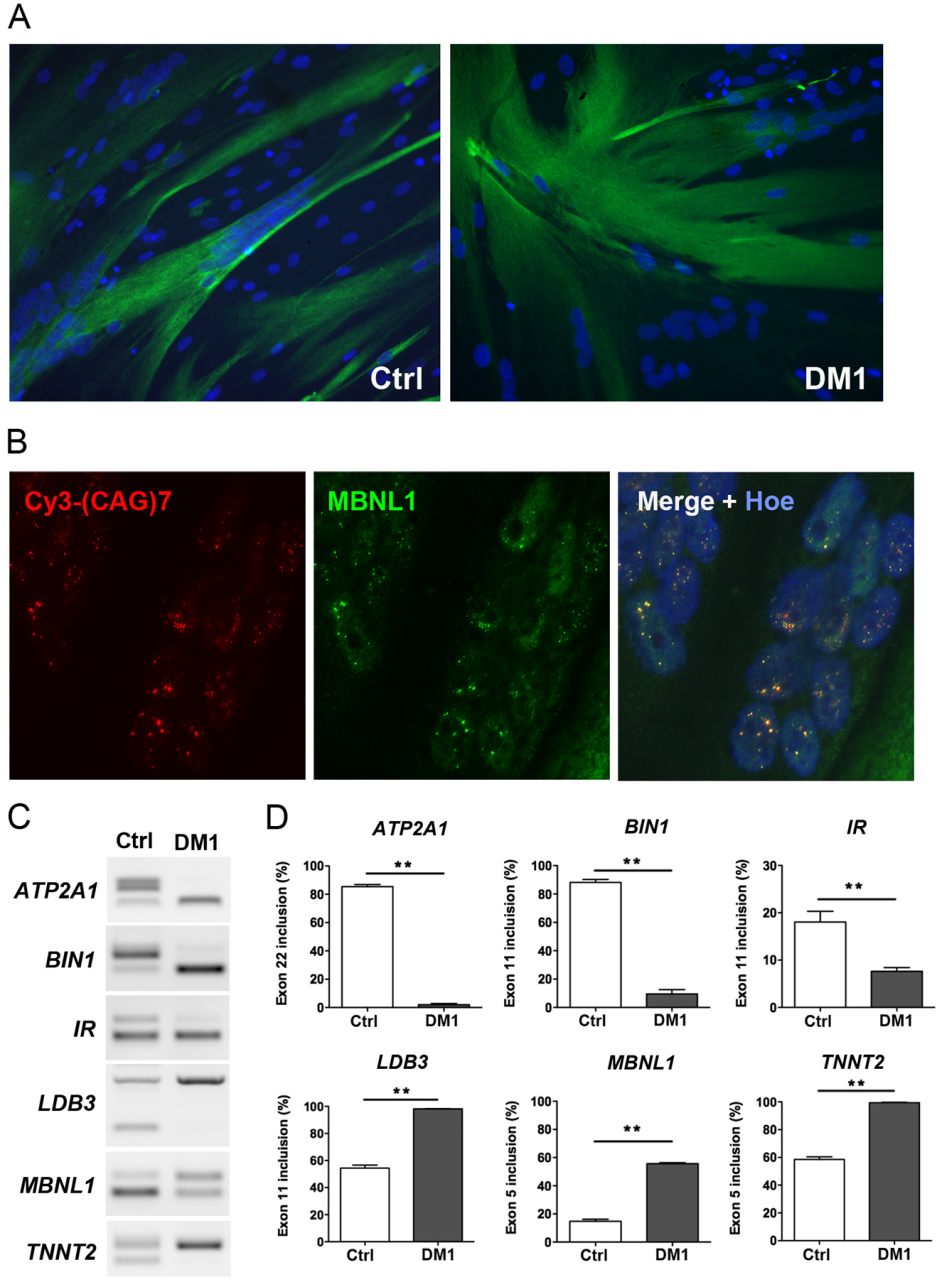
**Characterization of DM1 and Ctrl myotube cultures derived from conditional MyoD-converted fibroblast cell lines.** (A) Desmin (Desm) immunofluorescence staining (green) of myotube cultures derived from conditional MYOD1 converted Ctrl (left) and DM1 (right) fibroblast cell lines under permissive conditions to express MYOD1. (B) FISH using a Cy3-CAG7 probe (red) and MBNL1 immunostaining (green) showing the colocalization of MBNL1 with nuclear CUGexp-RNA aggregates in muscle converted DM1 immortalized fibroblasts. Hoe, Hoechst staining. (C,D) RT-PCR analysis and quantification of alternative splicing changes in *ATP2A1*, *BIN1*, *IR*, *LDB3*, *MBNL1* and *TNNT2* transcripts extracted from myotube cultures derived from converted DM1 and Ctrl fibroblast cell lines (*n*=4 for each condition). Data are expressed as mean±s.e.m. Comparison with Mann-Whitney test; **P<0.01.

An alternative method to produce human muscle cell lines, when muscle biopsies are not available, is to convert immortalized skin fibroblasts into multinucleated myotubes by forced expression of the myogenic regulator factor MYOD1 (Chaouch et al., 2009; Cooper et al., 2007; Lattanzi et al., 1998). Briefly, primary fibroblasts isolated from a skin biopsy are immortalized by the sole re-expression of TERT (Bodnar et al., 1998) before a subsequent transduction with lentiviral vectors containing an inducible Tet-on MYOD1 construct. Under non-permissive conditions, immortalized cells can proliferate indefinitely, but when supplemented with doxycycline, cells express MYOD1 that activates the myogenic program and their fusion into myotubes. Here, human primary fibroblasts isolated from skin biopsies obtained from a control individual (Ctrl) and from a DM1 patient, were used to establish immortalized myo-converted or transdifferentiated cell lines (hereafter referred to as transdifferentiated myotubes). Of note, both muscle and skin biopsies were obtained from the same DM1 patient. We showed that forced expression of MYOD1 following doxycycline supplementation leads to the formation of large multinucleated DM1 transdifferentiated myotubes comparable to Ctrl transdifferentiated myotubes (Fig. 3A). FISH and immunofluorescence experiments revealed that DM1 myotubes contain nuclear CUGexp-RNA aggregates that colocalized with MBNL1 splicing factor (Fig. 3B). Interestingly, DM1 transdifferentiated myotubes displayed splicing changes of many transcripts including *ATP2A1*, *BIN1*, *INSR*, *LDB3*, *MBNL1* and *TNNT2* (Fig. 3C,D) that are also misregulated in skeletal muscles of DM1 patients (Nakamori et al., 2013). Collectively, these results show that immortalized DM1 cell lines exhibit the characteristic features of DM1 pathology.

### DM1 muscle cell lines to assess therapeutic compounds

**Fig. 4.**
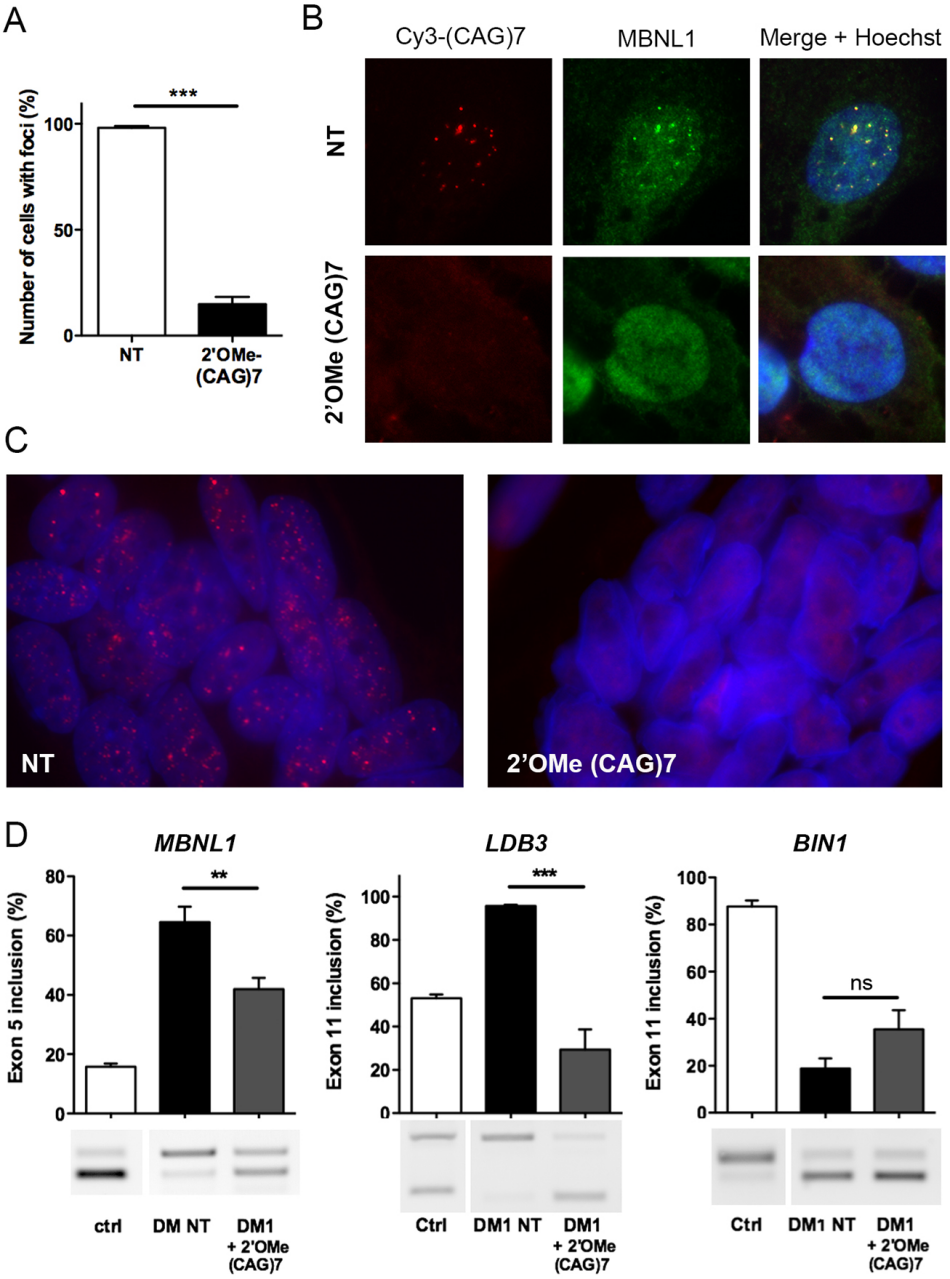
**(CAG)7-ASO reverses CUGexp-RNA toxicity in immortalized muscle DM1 cells.** (A) Percentage of immortalized DM1 myoblasts containing nuclear CUGexp-RNA aggregates (foci) following 2′OMe-(CAG)7 treatment (*n*=4). Data are expressed as mean±s.e.m. Comparison with Mann-Whitney test; ***P<0.001. (B) FISH immunofluorescence staining using a Cy3-(CAG)7 probe and an anti-MBNL1 antibody to detect nuclear foci (red) and MBNL1 (green), respectively, in immortalized DM1-cl5 myoblasts treated with 2′OMe-(CAG)7 ASOs compared with non-treated (NT) cells. (C) FISH experiment to detect CUGexp-RNA foci (red) in the nucleus (blue) of myotubes derived from converted DM1 fibroblast cell lines treated with 2′OMe-(CAG)7 ASOs. (D) Quantification of *MBNL1* exon 7, *LDB3* exon 11 and *BIN1* exon 11 splicing profiles determined by RT-PCR in myotubes cultures of converted Ctrl and DM1 fibroblast cell lines treated or not with 2′OMe-(CAG)7 ASOs (*n*=8, except for *BIN1* where *n*=6). Data are expressed as mean±s.e.m. Comparison with one-way ANOVA with Newman-Keuls post-test; **P<0.01, ***P<0.001; ns, not significant.

To determine whether these novel immortalized DM muscle cell lines represent relevant tools for *in vitro* screening or evaluation of therapeutic approaches, we used as a proof-of-concept, compounds known to neutralize the CUGexp-RNA toxicity in our model of DM1 muscle cell lines. We analyzed the effects of ASOs targeting the expanded CUG tract of mutant *DMPK* transcripts that inhibit the deleterious sequestration of MBNL1 splicing factor and reduce the number of nuclear CUGexp-RNA aggregates, thereby correcting DM1 splicing defects in DM1 mouse models (Mulders et al., 2009; Wheeler et al., 2009). In accordance with previous results obtained in DM1 myoblasts (Mulders et al., 2009; Pantic et al., 2016), 2′OMe-PT-(CAG)7 ASO treatment of DM1-cl5 myoblasts significantly decreased the number of nuclei with nuclear RNA aggregates (Fig. 4A), which was associated with a redistribution of sequestered MBNL1 proteins into the nucleoplasm (Fig. 4B). DM1 transdifferentiated myotubes were also treated with 2′OMe-PT-(CAG)7 ASO and a similar reduction in the number of nuclear CUGexp-RNA aggregates was observed after treatment (Fig. 4C) as previously shown by Larsen et al. (2011) in DM1 transdifferentiated myoblasts. Next, we tested the effects of 2′OMe-PT-(CAG)7 ASO treatment on splicing misregulation measured in DM1 transdifferentiated myotubes since only mild effects, if any, were described for this ASO both *in vivo* and *in vitro* (Mulders et al., 2009; Pantic et al., 2016). In accordance with these studies, splicing changes in *BIN1* exon 11, *ATP2A1* exon 22, *TNNT2* exon 5 and *INSR* exon 11 (Fig. 4D and data not shown) were not reversed. However, mis-splicing of *MBNL1* exon 5 and *LDB3* exon 11 were significantly corrected upon treatment with 2′OMe-PT-(CAG)7 ASO (Fig. 4D).

**Fig. 5.**
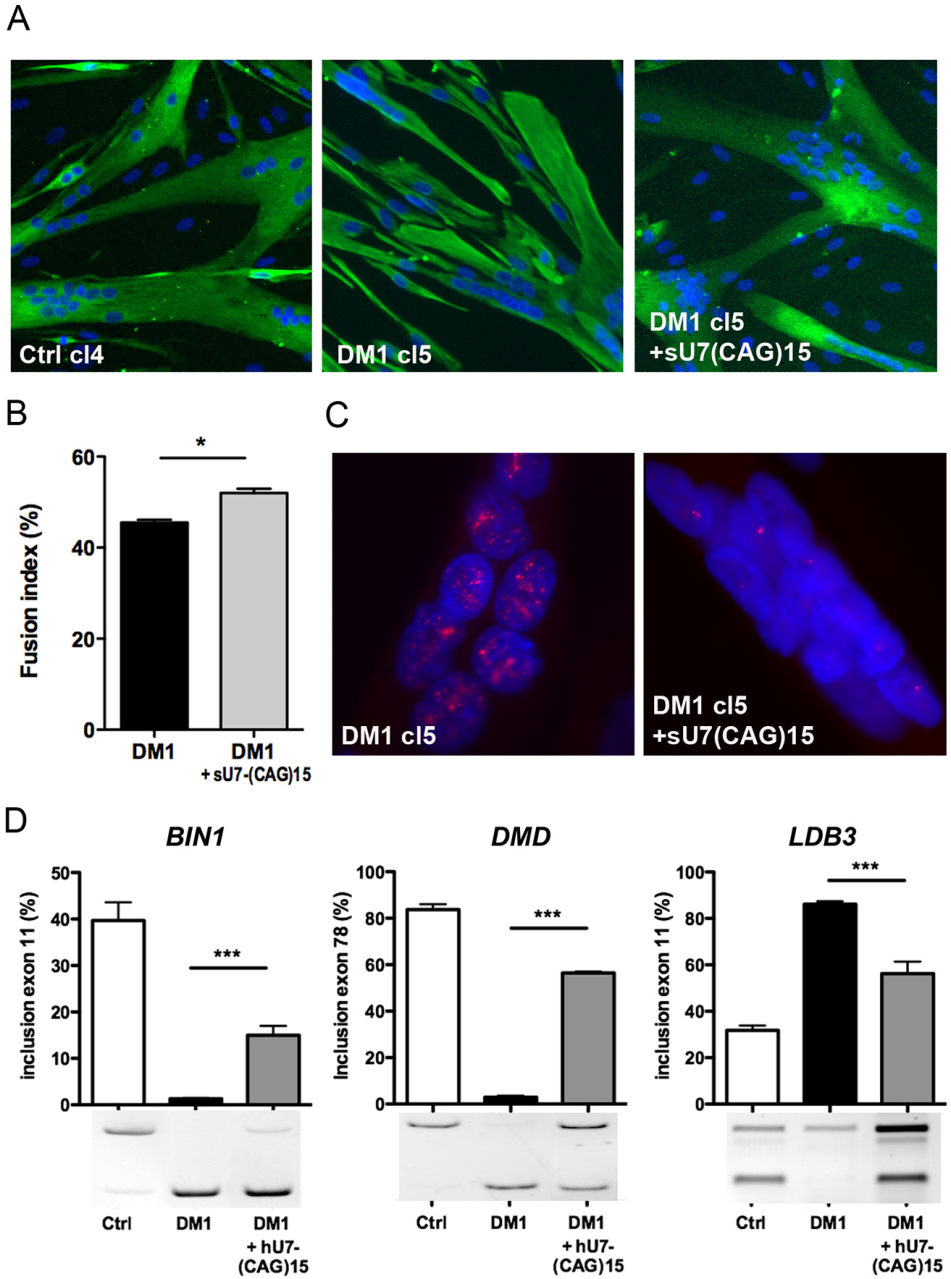
**hU7-(CAG)15 reverses CUGexp-RNA toxicity in DM1 myoblast cell lines.** (A) Differentiation of DM1-cl5 myoblast cell line expressing hU7-(CAG)15 antisense transcripts was assessed by desmin (green) and Hoechst (blue) immunostaining and compared with differentiated DM1-cl5 and Ctrl cells. (B) Fusion index of differentiated cultures of immortalized DM1 myoblasts transduced or not with hU7-(CAG)15 lentiviral vectors compared with Ctrl-cl4 (*n*=4 for each condition, >1000 nuclei counted per experiment). Data are expressed as mean±s.e.m. Comparison with Mann-Whitney test; *P<0.05. (C) Nuclear CUGexp-RNA aggregates in differentiated DM1-cl5 myoblast cell line expressing hU7-(CAG)15. (D) RT-PCR analysis of *BIN1* exon 11, *DMD* exon 78 and *LDB3* exon 11 splicing profiles in differentiated cultures of immortalized Ctrl-Cl4 and DM1-cl5 myoblasts transduced or not with hU7-(CAG)15 lentiviral vectors (*n*=6 for each condition). Data are expressed as mean±s.e.m. Comparison with one-way ANOVA with Newman-Keuls post-test; ***P<0.001.

A second antisense approach using a modified hU7-snRNA conjugated to a (CAG)15 sequence [hU7-(CAG)15] was then assessed in the immortalized DM1-cl5 myoblast cell line. Previously, we demonstrated the ability of hU7-(CAG)15 to silence CUGexp-RNA and correct splicing as well as differentiation defects in DM1 primary muscle cells (Francois et al., 2011). DM1-cl5 myoblasts were transduced with lentiviral vectors expressing the hU7-(CAG)15 construct and cultures were then switched to differentiation conditions. Myotubes formed by treated DM1-cl5 cells were similar to those observed in Ctrl-cl4 cells (Fig. 5A). The fusion index of differentiated DM1-cl5 myoblast cultures expressing hU7-CAG15 was increased compared with untreated differentiated DM1-cl5 myoblast cultures, and comparable to differentiated Ctrl-cl4 myoblast cultures (Fig. 5B). Moreover, expression of hU7-(CAG)15 antisense transcripts reduced the number of nuclear CUGexp-RNA aggregates in DM1-cl5 muscle cells (Fig. 5C) and partially corrected DM1-associated splicing changes for *BIN1* exon 11, *DMD* exon 78 and *LDB3* exon 11 (Fig. 5D).

**Fig. 6.**
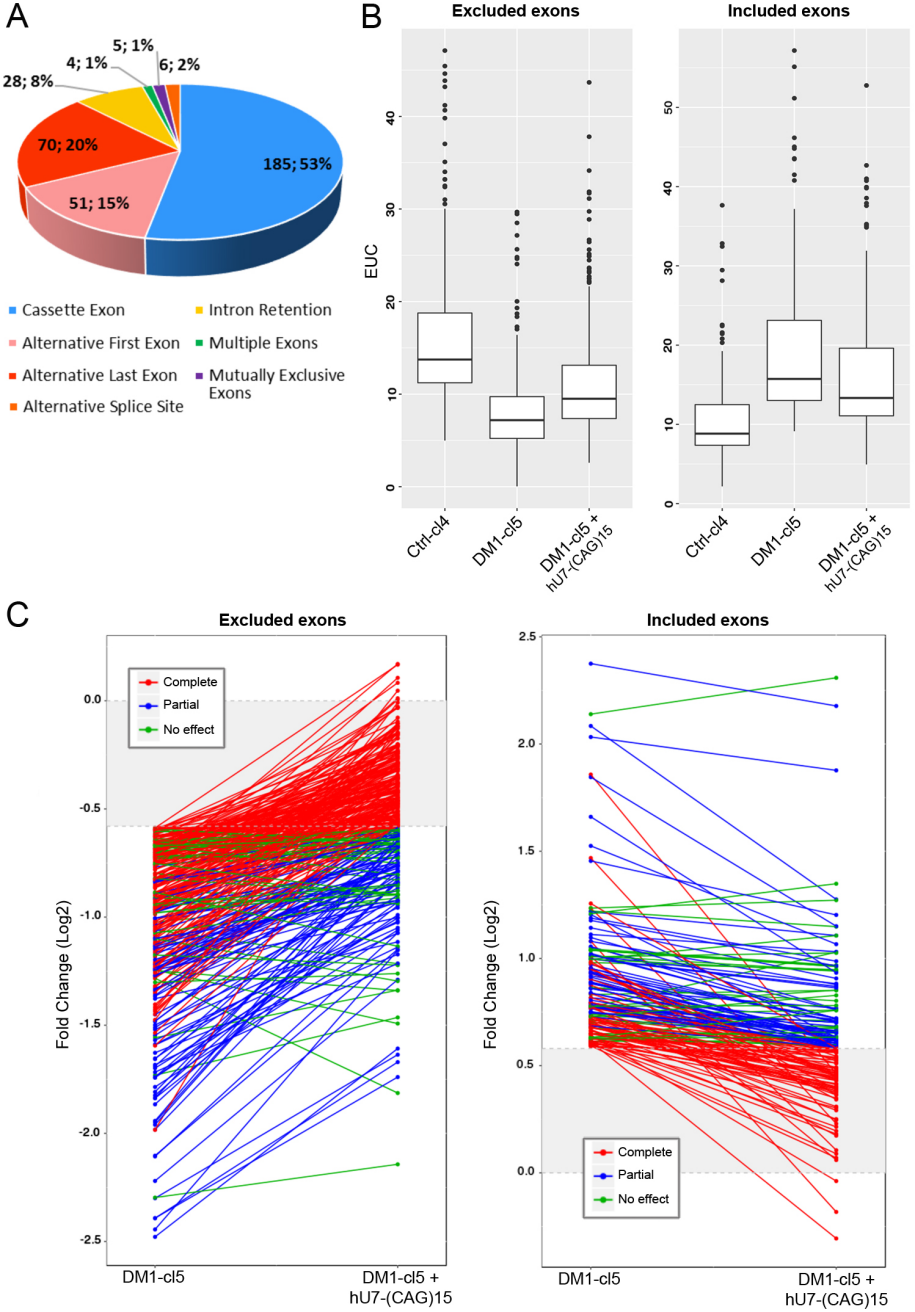
**Effect of hU7-(CAG)15 antisense oligonucleotide on global DM1-splicing changes.** Total RNA extracted from Ctrl-cl4, DM1-cl5 and hU7-(CAG)15-treated DM1-cl5 differentiated myoblasts analyzed with a paired-end RNA sequencing (RNA-seq). (A) Repartition of 349 mis-splicing events in DM1-cl5 compared with Ctrl-cl4 cells (number of events; percentage compared with total number of events). (B) Box plot representing splicing inclusion or EUC (exon usage coefficient, determined with DEX-seq software) of the 349 splicing events (exon_bin) regrouped in excluded exons (*n*=245, left panel) and included exons (*n*=104, right panel) in Ctrl-cl4, DM1-cl5 and DM1-cl5 treated with hU7-(CAG)15. In box plot, lower whisker represents smallest observation greater than or equal to lower hinge-1.5x interquartile range (IQR); lower hinge is 25% quantile, middle line is median; upper hinge is 75% quantile; upper whisker is largest observation less than or equal to upper hinge+1.5xIQR. (C) Analysis of individual exon_bin exclusion (left panel) or inclusion (right panel) event in DM1-cl5 cells compared with Ctrl-cl4 cells. More than 50% of events are fully normalized (red), 28% are partially restored (blue) and around 20% are not significantly modulated (green) by treatment with hU7-(CAG)15.

To evaluate the overall effect of hU7-(CAG)15-antisense on DM1-splicing changes, we performed paired-end RNA sequencing (RNA-seq) on total RNA extracted from Ctrl-cl4, DM1-cl5 and hU7-(CAG)15-treated DM1-cl5 differentiated myoblasts. Unfortunately, the RNA preparation method used in this experiment did not allow an efficient extraction of mutant *DMPK* transcripts carrying large CUG expansions. Bioinformatics analyses using DEX-seq software showed that 349 alternative splicing events (or exon_bin, listed in Table S1) were differentially regulated (fold change ≥1.5, adjusted *P*-value for multiple testing ≤0.1) in differentiated DM1-cl5 muscle cells when compared with Ctrl-cl4 differentiated muscle cells. Several mis-spliced events previously validated in skeletal muscles of DM1 patients such as *BIN1* exon 11, *CAPZB* exon 8, *DMD* exons 71 and 78, *GFPT1* exon 9, *KIF13A* exon 32, *ANK2* exon 21, *UBE2D3* exon 10 and *LDB3* exon 5 were also detected in the differentiated DM1-cl5 myoblast cell line. As shown in Fig. 6A, 53% of abnormal splicing events affect cassette exons and the vast majority of them (84%) are skipped single exon. Additional splicing events, including retention of alternative first or last exons and introns, were also found to be altered at 15%, 20% and 8%, respectively. Interestingly, the hU7-(CAG)15 antisense treatment affected 90% of mis-spliced events in differentiated DM1-cl5 myoblasts. The median level of rescue in treated DM1 cells was 41% for excluded events and 37% for included events (Fig. 6B). However, the level of correction in treated DM1 cells when compared with Ctrl cells was different for each mis-spliced event, as shown in Fig. 6C and listed in Table S1, with alternative splicing changes that were fully normalized (58% for excluded events and 51% for included events; red lines), partially corrected (28% for excluded or included events; blue lines) and not corrected or with less than 10% correction (14% for excluded events and 21% for included events; green lines). Globally, more than half (56%) of DM1-misregulated splicing events were no longer significantly altered in treated DM1 muscle cells when compared with Ctrl-cl4 muscle cells, confirming the partial neutralization of CUGexp-RNA toxicity by hU7-(CAG)15-antisense treatment.

## DISCUSSION

Development of relevant and robust cellular models for myotonic dystrophy in which the mutation is expressed within its natural context is essential to facilitate efforts that are pushed forward to identify new therapeutic compounds for this neuromuscular disorder. Among them, DM1-hES human embryonic stem cells and DM1 induced pluripotent stem (iPS) cells have been developed and provide powerful models as they have the ability to differentiate into many different cell types (Gao et al., 2016; Laustriat et al., 2015; Marteyn et al., 2011; Yanovsky-Dagan et al., 2015). Recently, protocols to differentiate human iPS cells in skeletal muscle cells have also been established; however, it remains a time-consuming procedure with variable efficiencies (Caron et al., 2016; Chal et al., 2015). In addition, primary muscle cell cultures derived from muscle biopsies of DM1 patients that reproduce characteristic molecular disease-associated features such as nuclear CUGexp-RNA aggregates have been used in many studies to either investigate molecular mechanisms or assess therapeutic compounds (Botta et al., 2013; Dansithong et al., 2005; Francois et al., 2011; Furling et al., 2001; Holt et al., 2007; Loro et al., 2010). However, one major limitation of this model, apart from the availability and accessibility of muscle biopsies from DM patients, is the limited proliferative capacity of human primary muscle cells due to replicative senescence (Bigot et al., 2009). To bypass these limitations and generate DM skeletal muscle cell lines, we immortalized human DM1 and DM2 primary myoblasts and selected clones from both cell lines that recapitulate phenotypic and molecular features similar to those found in primary DM muscle cell cultures.

### Immortalization of DM muscle cells

To prevent primary human skeletal muscle cells from entering replicative cellular senescence induced by telomere shortening and/or an active p16 pathway, it has been shown that re-expression of the catalytic subunit of the telomerase TERT to maintain telomere lengths associated with overexpression of CDK4, the natural ligand of p16 that inhibits its activity, are sufficient to extend the proliferative capacity and immortalize human primary myoblasts isolated from either healthy donors or muscular dystrophic patients (Mamchaoui et al., 2011). We previously demonstrated that the dual ectopic expression of TERT and CDK4 enables DM1 primary myoblasts to overcome pre-senescence and significantly extend their proliferative lifespan (Bigot et al., 2009). Immortalization of DM1 primary myoblast populations can also be achieved by combined ectopic expression of TERT, mutated CDK4(R24C) and cyclin D1 (CCND1), which increases the CDK4 kinase activity for efficient Rb hyperphosphorylation (Pantic et al., 2016; Shiomi et al., 2011). In the present study, transduction of primary myoblasts using lentiviral vectors allows integration of TERT and CDK4 constructs that contain selection markers and subsequently, establishment of stable cell lines. Based on their ability to differentiate and form myotubes, clones of immortalized Ctrl and DM myoblasts were isolated and the fusion index indicated that selected DM clones behave as parental primary muscle cell cultures.

### DM-associated molecular features in DM1 and DM2 muscle cell lines

At the molecular level, the presence of nuclear aggregates of either CUGexp- or CCUGexp-RNAs in immortalized DM1 and DM2 myoblasts, respectively, confirmed that both DM mutations were expressed in the corresponding muscle cell lines. Because the nuclear accumulation of mutant DM transcripts alters the function of RNA splicing factors such as MBNL1, misregulation of alternative splicing was assessed in immortalized DM muscle cells. Several abnormal splicing events that were validated in skeletal muscle of DM patients were indeed detected in differentiated DM1 cl5/cl6 myoblasts but not in differentiated DM2 clones, raising several questions. Independent of the nature of the different DM mutations, the size of the expanded repeat tract (2600 CTG in DM1 cells versus 4000 CCTG in DM2 cells) could also impact the pathophysiological mechanisms and ultimately explain the difference in splicing misregulation. To address this question, it would be interesting to have access to DM2 myoblasts with larger CCTG expansions, which can reach up to 11,000 repeats compared with the 5000 repeats in DM1. However, additional parameters may contribute to this difference, as DM2 pathology that affects mostly adults is usually milder than DM1, which affects adults and infants, and displays a severe congenital form (Liquori et al., 2001). Thus, the level of expression of C/CUG-expanded transcripts and the composition and/or the structure of the expanded tract itself (CUG versus CCUG) should also influence the behavior of the ribonucleoprotein complexes and sequestered/associated proteins. Although MBNL1 colocalized with both C/CUGexp-RNA aggregates, the extent of sequestered MBNL1 and subsequently, the levels of remaining free and functional protein may be different in DM1 and DM2 cells, particularly if the dynamics of accumulation of MBNL1 into CUGexp- and CCUGexp-RNA aggregates is not the same. Moreover, additional molecular alterations and/or modifiers that have been described in DM1 muscles could also be involved in this difference. Finally, altered myogenic differentiation and fusion of DM1 myoblasts might also interfere with the level of alternative splicing changes. However, this seems unlikely because similar splicing defects were detected in DM1 transdifferentiated myotubes overexpressing MYOD1 that display similar myogenic differentiation to Ctrl transdifferentiated myotubes, as previously described (Larsen et al., 2011). Furthermore, MYOD1 overexpression has also been shown to rescue the differentiation defects observed in myoblasts expressing CUGexp-RNA and associated with a compromised MYOD1 level (Amack et al., 2002). Overall, our data demonstrate that the molecular hallmarks associated with the toxic RNA gain-of-function mechanism, such as nuclear RNA aggregates and splicing defects are retained in immortalized DM1 muscle cells, and represent valuable readouts to assess the efficacy of therapeutic compounds.

### Therapeutic approach assessment

To confirm that our new immortalized DM1 muscle cell lines are reliable tools for therapeutic compound screenings, we tested antisense approaches that have shown their efficacy to reverse CUGexp-RNA toxicity (Francois et al., 2011; Gonzalez-Barriga et al., 2013; Mulders et al., 2009; Wheeler et al., 2009). We found that CAG antisense treatments that target the expanded CUG tract such as hU7-(CAG)15 or ASO-(CAG)7, decrease the number of nuclear CUGexp-RNA aggregates, which has previously been shown to correlate reduced levels of CUGexp-*DMPK* mRNA, and correct splicing defects in our immortalized DM1 muscle cells. Of note, the normalization of DM splicing changes following CAG-antisense treatment in immortalized DM1 muscle cell models was not reported before (Pantic et al., 2016; Larsen et al., 2011). Our results are in accordance with an increasing body of evidence, indicating that functional loss of MBNL1 due to its sequestration into nuclear RNA aggregates results in misregulation of alternative splicing (Cardani et al., 2006; Lin et al., 2006; Mankodi et al., 2001; Miller et al., 2000). As the number of nuclear CUGexp-RNA aggregates sequestering MBNL1 in immortalized DM1 muscle cells is greatly reduced by antisense treatment, we hypothesize that the pool of free and functional MBNL1 is increased leading ultimately to the rescue of abnormal splicing events in DM1 cells. Accordingly, 90% of the 349 mis-spliced events identified by RNA-seq analysis in DM1-cl5 differentiated myoblasts were affected by antisense treatment, and more than half of them were normalized in treated-DM1 cells compared with control cells. Because CUGexp-RNA toxicity can be reversed by antisense compounds targeting CUG-expanded transcripts, we propose that both nuclear RNA aggregates and splicing defects represent helpful molecular readouts to screen and evaluate therapeutic compounds in our DM1 muscle cell lines.

In conclusion, we describe the development and characterization of innovative new immortalized DM muscle cell lines, as well as their potential use to screen therapeutic strategies and compounds. We show that selected clones of immortalized DM1 and DM2 myoblasts behave like parental primary myoblasts isolated from patient muscle biopsies, and contain nuclear aggregates of RNA containing expanded repeat tract, which are typical molecular features associated with the expression of DM mutations. In addition, RNA-seq analysis showed that more than 300 splicing events are altered in immortalized DM1 muscle cells compared with immortalized Ctrl muscle cells. Lastly, as a proof-of-concept, we were able to reverse CUGexp-RNA toxicity associated with nuclear RNA aggregates and abnormal splicing changes in immortalized DM1 muscle cells using CAG-antisense treatments. Therefore, immortalized DM muscle cell lines represent new valuable models for the assessment of potential therapeutic compounds.

## MATERIALS AND METHODS

### Cell culture

Muscle and skin biopsies were obtained from the MyoBank-AFM bank of tissues for research, a partner in the EU network EuroBioBank, in accordance with European recommendations and French legislation. Muscle biopsies were obtained from a semitendinosus muscle of a 25-year-old unaffected male individual (Ctl), a quadriceps muscle of a 53-year-old female patient suffering from DM2 (4000 CCTG in the muscle) and a gastrocnemius muscle of an 11-year-old female patient suffering from an infantile DM1 form (1300 CTG in the blood). Primary cells were isolated from skeletal muscle or skin biopsies as described previously (Chaouch et al., 2009; Edom et al., 1994). Myoblasts are cultivated in a growth medium consisting of a mix of 199:DMEM (1:4 ratio; Life Technologies) supplemented with 20% FBS (Life Technologies), 50 μg/ml gentamycin (Life technologies), 25 µg/ml fetuin, 0.5 ng/ml bFGF, 5 ng/ml EGF and 0.2 µg/ml dexamethasone (Sigma-Aldrich). Fibroblasts are cultivated in a DMEM growth medium supplemented with 15% FBS and 50 μg/ml gentamycin. Myogenic differentiation is induced by switching confluent cell cultures to DMEM supplemented with 5 µg/ml insulin for myoblasts, and a additional 4 µg/ml of doxycycline (Sigma-Aldrich) for immortalized MYOD1-inducible fibroblasts.

### Immortalization of primary myoblast and fibroblast

Immortalization and selection processes were done as previously described (Chaouch et al., 2009; Mamchaoui et al., 2011). Briefly, *TERT* and *Cdk4* cDNAs were cloned into different lentiviral vectors containing puromycin and neomycin selection markers, respectively. Myoblasts were transduced with both *TERT* and *Cdk4* lentiviral vectors while fibroblasts were transduced with *TERT* vectors only, with a MOI of 3-5 in the presence of 4 µg/ml of polybrene (Sigma-Aldrich). Transduced cell cultures were selected with puromycin (0.2 μg/ml) and/or neomycin (0.3 mg/ml) for 8 days. Transduced cells were seeded at clonal density and selected individual clones were isolated from each population. For conditional expression of MYOD1, immortalized fibroblasts were transduced with lentiviral vectors expressing murine *Myod1* cDNA under the control of a Tet-on inducible construct (Barde et al., 2006).

### Fluorescent *in situ* hybridization and immunofluorescence

FISH experiments were done as previously described (Francois et al., 2011) using a Cy3-labeled 2′OMe (CAG)_7_ or (CAGG)_5_ probe. Immunofluorescence (IF) experiments were done as previously described (Klein et al., 2008) using anti-desmin antibody (D33 clone; 1:50, DAKO, M0760). Combined FISH-IF experiments were done using a Cy3-labeled (CAG)_7_ probe and a monoclonal anti-MBNL1 antibody (MB1a; gift from G. Morris, (Institute for Science and Technology in Medicine, Keele University, UK, 1:200) followed by a secondary Alexa Fluor 488-conjugated goat anti-mouse (1:500, Life Technologies, A-11029) antibody. Pictures were captured using an Olympus BX60 microscope and Metamorph software (Molecular Devices), and processed with Adobe Photoshop software. Fusion index of differentiated muscle cell cultures was calculated as a percentage of nuclei inside myotubes (>2 myonuclei) to the total number of nuclei in desmin-positive cells.

### RNA isolation and RT-PCR

Total RNAs were isolated using TRIzol reagent (Life Technologies) according to the manufacturer's protocol. One µg of RNA was reverse transcribed using M-MLV first-strand synthesis system according to the manufacturer's instructions (Life Technologies) in a total volume of 20 µl. One microliter of cDNA preparation was subsequently used for PCR according to standard procedures (ReddyMix, Thermo Scientific). Primers used to analyze the splicing profiles of the different transcripts are indicated in Table 1.

**Table 1.**
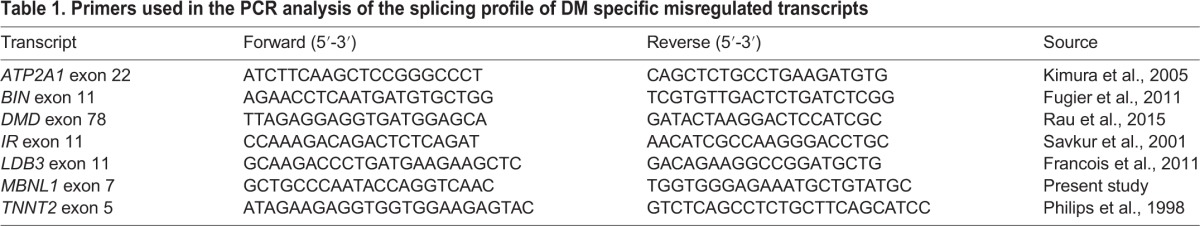
**Primers used in the PCR analysis of the splicing profile of DM specific misregulated transcripts**

### Antisense treatments

For hU7-(CAG)15 expression, 1.5×10^5^ immortalized DM1 myoblasts were transduced with 1×10^7^ vg/ml of hU7-(CAG)15 lentiviral vectors. Transduction was performed twice for 4 h in the presence of 4 µg/ml of polybrene (Sigma-Aldrich) and the transduced cells were grown at least 1 week before differentiation and/or analyses. For ASO treatment, proliferating immortalized DM1 myoblasts or myotubes-converted DM1 fibroblast cell lines (after 4 days of differentiation) were transfected with 2 µg/ml of 2-OMe-PT-(CAG)7 ASOs using RNAi Max transfection reagent (Life Technologies) according to the manufacturer's protocol. Cells were harvested for analysis 24 h after transfection.

### Southern blot analysis

Ten micrograms of genomic DNA were digested by *Hin*dIII and *Sac*I and subjected to 0.7% agarose gel electrophoresis for 48 h with 1 kb Ladder (New England Biolabs) as molecular size markers. After denaturation (1 M NaOH) and neutralization (1 M Tris-HCl, 3 M NaCl, pH 8.3), DNA was transferred to a Hybond-N membrane in 6× SSC. Hybridization was carried out using a pM10M6 ^32^P-labeled probe, a 1.4 kb *Bam*HI fragment containing the CTG repeat tract (Brook et al., 1992; Furling et al., 2001). The membrane was then washed and analyzed by autoradiography. The size of the CTG repeat was measured using ImageJ software and the value was determined from the middle of the smear. DNA isolated from control cells were used as a negative control.

### RNA sequencing and analysis

Total strand RNA-seq was performed by the Centre National de Génotypage (Institut de Génomique, CEA). After complete RNA quality control on each sample (quantification in duplicate and RNA6000 Nano LabChip analysis on Bioanalyzer from Agilent), libraries were prepared using the TruSeq Stranded Total RNA with Ribo-Zero Kit (Illumina), with an input of 1 µg. Library quality was checked by Bioanalyzer analysis, and sample libraries were pooled before sequencing to reach the expected sequencing depth. Sequencing was performed on an Illumina HiSeq2000 as paired-end 100 bp reads, using Illumina TruSeq V3 reagents

RNA-seq reads were trimmed using Trimmomatic (Bolger et al., 2014) and aligned to human transcriptome using Tophat2 (Kim et al., 2013). Raw count tables were calculated using HT-seq (Anders et al., 2012) and differential splicing events between two groups of samples were identified using DEX-seq [FDR≤0.1, abs(log2 fold change)≥0.58] (Anders et al., 2012). A minimum read coverage of 30 was required in at least one condition to performed analysis at exon level. After analysis, a manual curation was performed to remove remaining technical artefacts and precisely annotate alternative splicing events.

### Statistical analysis

All group data are expressed as mean±s.e.m. Between groups, the comparison was performed by Mann-Whitney test or one-way ANOVA with Newman-Keuls post-test using Prism 5 software (GraphPad). Differences between groups were considered significant when *P*<0.05 (*P<0.05; **P<0.01; ***P<0.001).
